# Relating annual increments of the endangered Blanding's turtle plastron growth to climate

**DOI:** 10.1002/ece3.1065

**Published:** 2014-04-22

**Authors:** Monik G Richard, Colin P Laroque, Thomas B Herman

**Affiliations:** 1 Department of Biology, Acadia University33 Westwood Avenue, Wolfville, Nova Scotia, Canada, B4P 2R6; 2 Department of Soil Science, University of Saskatchewan51 Campus Drive, Saskatoon, Saskatchewan, Canada, S7N 5A8

**Keywords:** Blanding's turtle, *Emydoidea blandingii*, plastron growth increments, temperature, dendrochronology, climate change

## Abstract

This research is the first published study to report a relationship between climate variables and plastron growth increments of turtles, in this case the endangered Nova Scotia Blanding's turtle (*Emydoidea blandingii*). We used techniques and software common to the discipline of dendrochronology to successfully cross-date our growth increment data series, to detrend and average our series of 80 immature Blanding's turtles into one common chronology, and to seek correlations between the chronology and environmental temperature and precipitation variables. Our cross-dated chronology had a series intercorrelation of 0.441 (above 99% confidence interval), an average mean sensitivity of 0.293, and an average unfiltered autocorrelation of 0.377. Our master chronology represented increments from 1975 to 2007 (33 years), with index values ranging from a low of 0.688 in 2006 to a high of 1.303 in 1977. Univariate climate response function analysis on mean monthly air temperature and precipitation values revealed a positive correlation with the previous year's May temperature and current year's August temperature; a negative correlation with the previous year's October temperature; and no significant correlation with precipitation. These techniques for determining growth increment response to environmental variables should be applicable to other turtle species and merit further exploration.

## Introduction

For over 100 years, dendrochronology, the study of annual growth increments in trees, has explored the relationship between tree growth and environmental signals, such as temperature, and precipitation, which affect tree growth and are consequently reflected in ring widths (Fritts 1976; Speer 2010). With the advancement of computer-based tools that allow more robust statistical analysis of tree-increment datasets, dendrochronology has been advancing rapidly and revolutionizing the study of annual growth increments in other species. Within the last decade, for example, annual increments in corals (Dodge and Lang 1983; Guzman et al. 2008), Arctic dwarf shrubs (Johnstone and Henry 1997; Rayback and Henry 2006), fish otoliths (Black et al. 2005), and mollusks (Black et al. 2008; Rypel et al. 2008) have produced good proxy relationships to these organisms' environments using established dendrochronological methods.

Measuring annual growth increments from the plastron of turtles has been used commonly for growth modeling, aging, and population dynamic studies (see Hailey and Coulson (1999) and Wilson and Tracy (2003) for extensive lists), but apart from one unpublished study on wood turtles (Robichaud 2006), the measured widths of an individual's annual increments have not been related to environmental parameters. Understanding the relationship between annual growth increments and climate helps us to better understand limiting or driving growth factors and makes possible both paleoenvironmental reconstructions (cf. geoduck clams, Strom et al. (2004)) and predictions of the effects of climate change on a species using available future climate models (Laroque and Smith 2005; Robichaud 2006).

Annual plastron growth increments are clearly visible on immature Blanding's turtles (*Emydoidea blandingii*) (Lefebvre et al. 2011; Richard 2012) and are easily recorded in scans (Huang et al. 2008) or photographs. As the turtles age, growth increments on their plastrons begin to erode from abrasion during normal movements, making observation of increments in adults unreliable. The widths of these plastron growth increments vary from year to year, and so provide a potential window to better understand ecological factors driving their overall growth.

The goal of this research was twofold: first, to determine whether existing dendrochronological methods could be adapted to create a cross-dated series of growth rings and second to look for any relationship between the growth ring series and environmental parameters. Because turtles are ectothermic, we hypothesized that turtle annual growth increments would exhibit a significant response to the climatic variables of temperature and precipitation.

### Study species

Nova Scotia's Blanding's turtle (*E. blandingii*) population is geographically isolated in the southwestern part of this peninsular province and at the northeastern periphery of the species range (Mockford et al. 1999). The main range of the Blanding's turtle is south of the Great Lakes and includes southern parts of the Canadian provinces of Ontario and Quebec as well as an isolated population in Nova Scotia (Herman et al. 1995). In the United States, its main range occurs in Indiana, Missouri, Illinois, Iowa, Minnesota, Nebraska, and Wisconsin with isolated populations in New York, Maine, and Massachusetts (Herman et al. 1995). Due to its small population size, isolation, and specific habitat requirements, the species is listed as endangered in Canada and by the International Union for Conservation of Nature (van Dijk and Rhodin 2011). While it has protection in several states, the Blanding's turtle has no federal status in the United States. The Nova Scotia Blanding's turtle recovery team estimates the entire provincial population comprises approximately 350 adults (Arsenault 2011). Although there are no accurate estimates of the total numbers of immature turtles in the wild, the Nova Scotia Blanding's turtle recovery team, estimates they, represent roughly 40% of the total Nova Scotia Blanding's population.

In general, Blanding's turtle habitat consists of bogs, fens, narrow watercourses, and lakes with overhanging vegetation (Newton and Herman 2009; Kydd 2010). Recent work on immature turtles revealed that this age class seems to prefer more sheltered *Sphagnum*-rich coves, meadows, and brooks (Arsenault 2011). The ecology of wild immature Blanding's turtles is not well understood in the Nova Scotia populations (Herman et al. 1995; McNeil 2002; Arsenault 2011), as little is known about their natural history past the hatchling stage, until they reach adulthood.

Blanding's turtles in Nova Scotia are long-lived (over 80 years; Herman et al. 1995) and may take up to 25 years to mature, an older age than those in the main part of the range (Lefebvre et al. 2011). In turtles, immature survival and adult survival have a greater impact on population stability than egg and hatchling survival (Congdon et al. 1993; Heppell et al. 1996 Enneson and Litzgus 2008; ). Ecological variables driving immature survival are likely related to habitat availability, food availability, climate, competition, and predation (Lefebvre et al. 2011).

### Study sites

**Figure 1:**
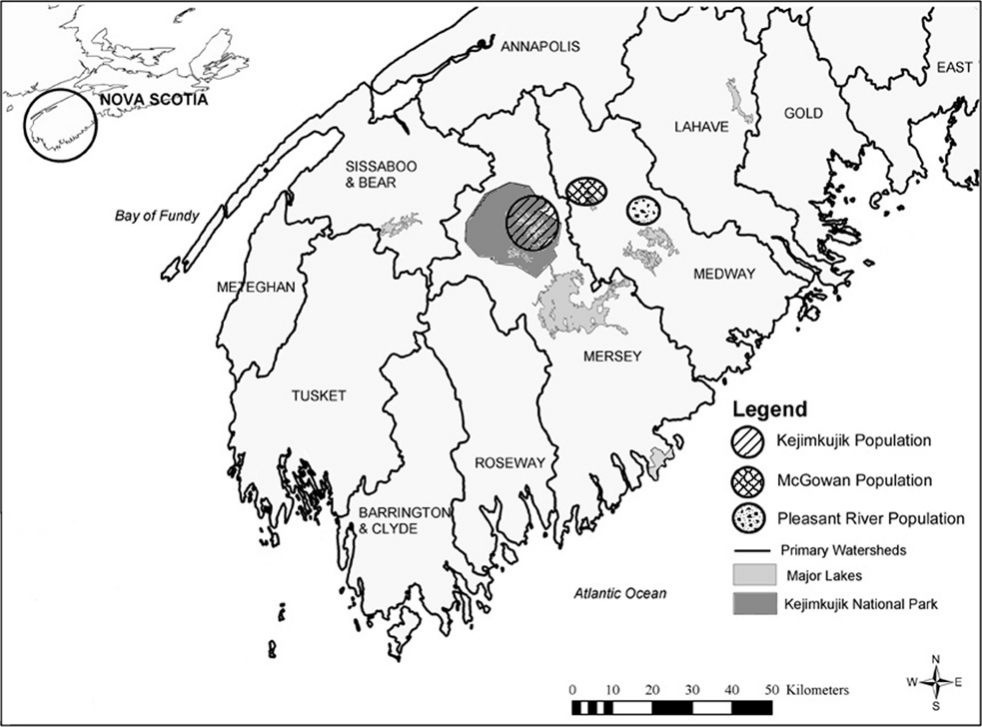
Map of southwest Nova Scotia showing study sites in three populations of NS Blanding's turtle (Used with permission from Mockford et al. 2005).

All data for this research were obtained in the areas of Kejimkujik National Park and National Historic Site (KNP), McGowan Lake (ML), and Pleasant River (PR) (Fig. 1) genetically distinguishable subpopulations (Mockford et al. 2005) in interior southwest Nova Scotia. Dominated by Acadian forest, bogs, fens, watercourses, and oligotrophic lakes, KNP is located in the Mersey watershed (44°15′ to 44°30′N, 65°00′ to 65°30′W) and is the only federally protected habitat for Blanding's turtles in the province. ML is located in a part of the Medway watershed (44°25′ to 44°28′N, 65°05′ to 65°02′W) where significant turtle habitat has been protected, but the remaining unprotected zones are heavily impacted by residences, farmlands, and forestry. PR is also located in the Medway watershed (44°24′ to 44°27′N, 65°53′ to 65°56′W), contains no legally protected habitat, and remains impacted by human presences such as residences, farmlands, pastures, tree farms, and forestry activities. Effective stewardship and outreach have occurred at all three sites (Caverhill 2006).

## Materials and Methods

### Turtle surveying and plastron scanning

Blanding's turtle researchers collaborated to sample immature turtles using a combination of live trapping and visual surveying in all three study sites from May to November 1998–2007. Visual surveys were conducted using repeatable transects in wetlands and riparian areas. Trapping was conducted using aquatic hoop-net traps specially designed for livetrapping Blanding's turtles in their habitats (Caverhill 2003). Immature turtles captured for the first time each year were transported in an open plastic container with approximately 10 cm of native water to one of two field stations. Here, the plastron was scanned at a minimum resolution of 360 dpi by centering the plastron of the turtle vertically and horizontally on a standard color flatbed scanner without a marker to indicate scale. Instead, overall plastron width and length were measured using calipers. Each turtle was released at its capture location within 24 h.

**Figure 2:**
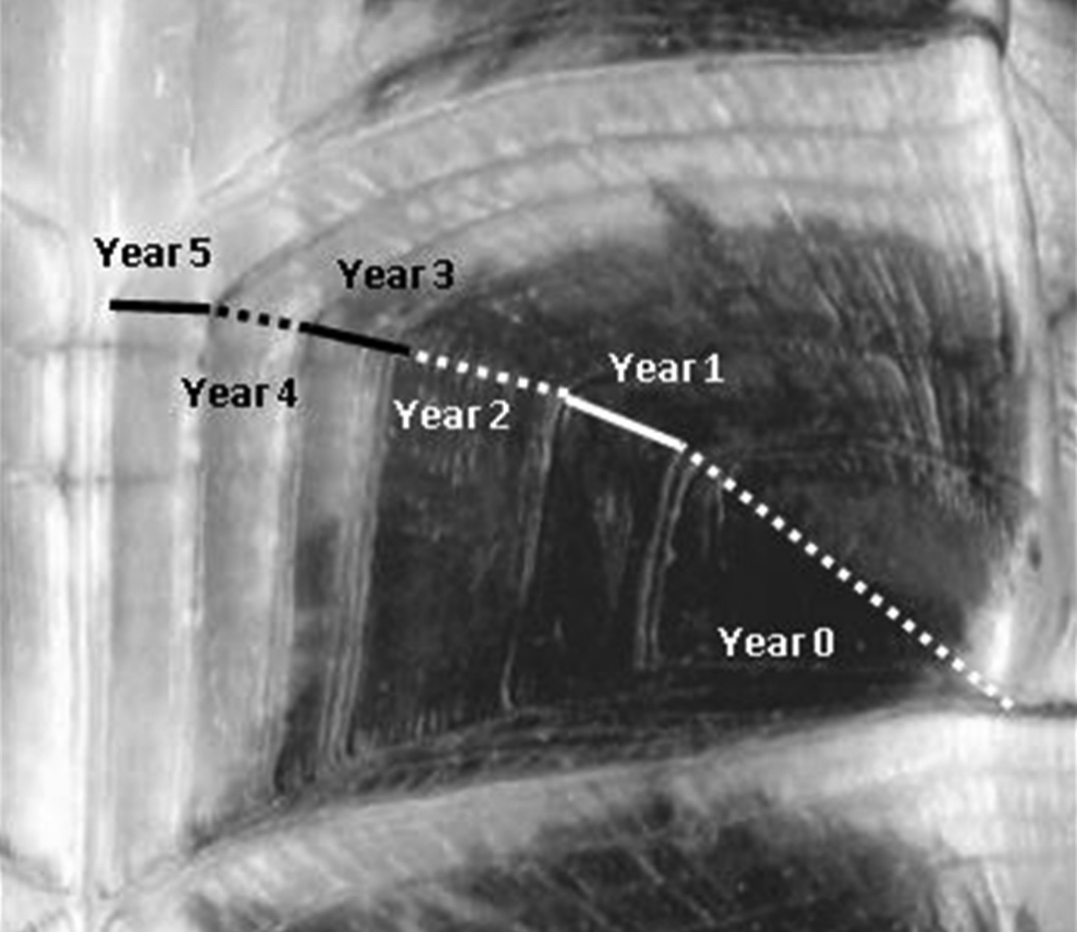
Partial Blanding's turtle plastron scan showing measurement location at diagonal line of growth (approximate measures).

Blanding's turtle plastrons comprise six pairs of scutes. Each scute pair is symmetrical, but successive scutes are asymmetrical (Huang et al. 2008). Width measurements were taken along a diagonal line bisecting the scute (Fig. 2). The current year of growth, because it could be an incomplete growth record, was measured, but omitted from further steps in this study. We used *ImageTool* version 3.0 (S. B. Dove 2002), a free image analysis software tool, that uses pixels to measure the widths of the increments digitally for third scute down from the head on the left side. To convert measurements from pixels to mm, a conversion factor was calculated for each individual turtle by averaging the field measurements for maximum plastron width and maximum plastron length obtained with calipers.

### Cross-dating

Cross-dating is a pattern-matching process whereby individual growth increments are cross-referenced in order to determine whether the widths of one annual increment, in our case within a specific plastral scute, correlate with the same growth-year increment on the same scute of another turtle. Cross-dating was used to verify/assign the correct calendar year to each increment (Grissino-Mayer 2001). Cross-dating can be successful only when growth is recorded at regular intervals, in this case annually (Lefebvre et al. 2011; Richard 2012), and when a common environmental response signal influences growth in a similar manner for all individuals within a population.

To ensure that each growth increment was properly assigned to the right calendar year, we first conducted a visual review of our measurements seeking commonalities in wide increments and narrow increments in specific years. Three narrow pointer years were consistently seen, 1996, 1992, and in the older sequences, 1986. Next, the measurement data were analyzed by COFECHA (Version 6.06P; Holmes et al. 1986), a standard statistical program used in dendrochronology to assess the quality of cross-dating individual measurement series relative to one another (Grissino-Mayer 2001). When a measurement series illustrates year-to-year variations in increment widths, individuals of different ages can potentially be overlapped by cross-dating their patterns. COFECHA uses a segmented time series correlation technique to assess the quality of overlap in a given measurement series (Grissino-Mayer 2001). COFECHA creates an output file that assigns a correlation value to each segmented time series and flags individual chronologies that do not correlate well with the pattern created by the overall assembled series (Grissino-Mayer 2001). It then indicates suggested changes in dating, if needed, based on locations where the segmented series might fit better. It conducts this goodness-of-fit test using correlation values to help the user determine whether measurement or dating errors may have occurred (Grissino-Mayer 2001).

COFECHA also provides some overall series statistics, giving values for intercorrelation, average mean sensitivity, and average unfiltered autocorrelation. Intercorrelation values describe the strength of the relationship of a given turtle's increment pattern to the overall pattern of all of the turtles combined. The mean sensitivity value is a relative measure of increment width change from 1 year to the next. A high mean sensitivity value indicates that dramatic variation can occur from one ring to the next, while a low sensitivity value indicates complacent increments, all of similar width. The unfiltered autocorrelation value indicates how much the previous year's growth predetermines the growth of the increment in the given growth year, with high values indicating a higher dependence on the previous year's conditions.

### Detrending the growth increment series

Blanding's turtle plastron growth increments follow a von Bertalanffy growth curve (Huang et al. 2008). Therefore, some type of negative exponential detrending was deemed necessary to standardize the growth increments from one turtle to the next, and then compile the measurements into one common chronology. To accomplish this, we used the dendrochronology program ARSTAN (version 41d; Cook and Peters 1981), which utilizes user-defined options to detrend and standardize time series measurements. Standardization eliminates the units of measurement and results in a unitless series with a mean of one. All other standardized increments vary above (wider than average) or below (narrower than average) the mean. In our case, we used single detrending with a negative exponential curve function to correspond with the turtle's growth model. ARSTAN is then able to apply a robust averaging function to create a single master chronology from all of the turtle measurements after detrending (Cook and Holmes 1986). We chose the “standard” output chronology to allow any natural autocorrelation in the overall time series to be included in the final master chronology series. We used this master chronology for all subsequent analyses.

### Climate response analysis

As ectotherms, Blanding's turtles bask to absorb environmental heat necessary for metabolic functions, so we looked at air temperature and precipitation variables in order to attempt to relate them to the master chronology of annual growth increments. We used DENDROCLIM2002 (version 1.0.0.1; Biondi and Waikul 2004) to determine statistically whether monthly temperature and precipitation variables relate to our turtle master chronology. DENDROCLIM2002, another commonly used dendrochronology program, uses bootstrapped confidence intervals to estimate the significance of both correlation and response function coefficients for temperature and precipitation variables (Biondi and Waikul 2004). A correlation function relates the turtle increment chronology to the monthly temperature or precipitation variable (Biondi 1997), where coefficients are univariate estimates of Pearson's correlation (Biondi and Waikul 2004).

We ran the master chronology in DENDROCLIM2002 using mean monthly air temperature and mean monthly precipitation data from the Greenwood, Nova Scotia, Environment Canada climate station (station ID #8202000), available online through Environment Canada's website (http://www.ec.gc.ca). The Greenwood station was selected as being the geographically closest set of normalized temperature and precipitation data for the years corresponding to our growth increments. We selected a continuous window of data from previous year March to current year November, corresponding with the turtles' active season (Newton and Herman 2009; Kydd 2010), and ran an analysis on temperature and precipitation independently due to degrees of freedom limitations.

## Results

### Cross-dating

We successfully cross-dated the measurement sequences for 80 of our original 98 turtles for a total time span of 33 years (1975–2007). As seen in Table 1, the cross-dated chronology had a series intercorrelation of 0.441 (significant using a 30-year window at the 99% confidence interval), an average mean sensitivity of 0.293, and average autocorrelation of 0.377. Eighteen turtle measurement sequences were discarded either because the turtles were very young, image quality was poor, or deformities were present in the scute increments. Growth-measurement sequences ranged from a low of 4 years to a high of 27 years with a period from 1982–2006 as the time frame in which a sample depth of two or more sequences overlapped in time.

**Table 1:** COFECHA results used to cross-date 80 Blanding's turtle growth increment chronologies

Number of chronologies cross-dating	Time span represented	Average series intercorrelation^1^	Mean sensitivity^2^	Average series autocorrelation^3^	Average length of series	Period with 2 or more chronologies
80 of 98	1975–2007 (33 years)	0.441	0.293	0.377	11 years	1982–2006

1 All values over 0.4226 significant at the 99% level.

2 Mean sensitivity is the measure of the relative change in increment width from 1 year to the next.

3 Autocorrelation indicates how much the previous years growth predetermines the growth of the increment in the given year.

### Detrending the growth increment series

**Figure 3:**
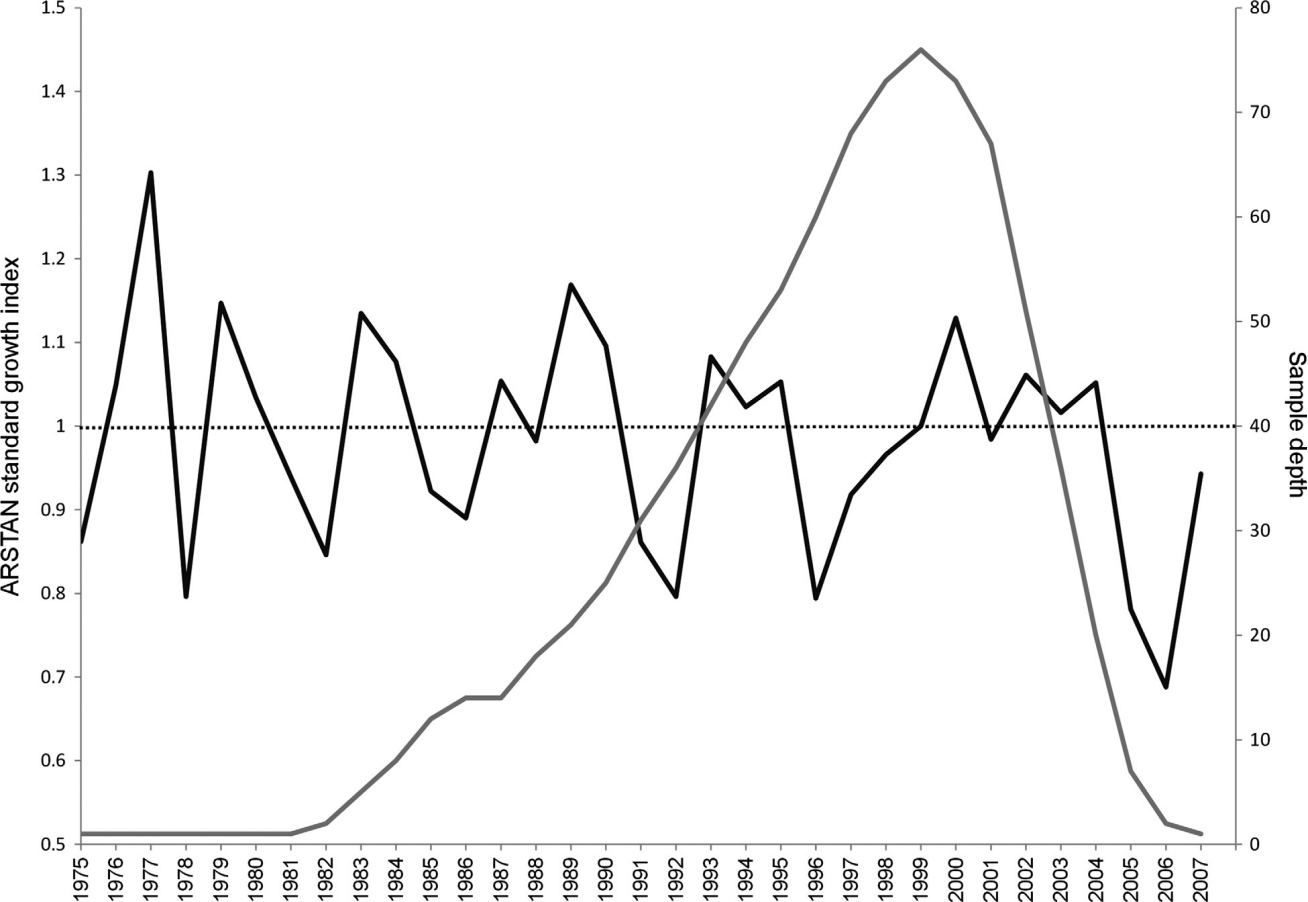
Master chronology of 80 Blanding's turtles using ARSTAN standard growth index (black line) and showing sample depth (gray line). Sample depth shows the variation in the total number of individuals used to compute each year of the master chronology.

With these 80 cross-dated measurement series, we were able to produce a master chronology (Fig. 3) in ARSTAN. The master chronology illustrates the average growth value as one, and values above and below one represent wider and narrower growth increments within their given years respectively. The master chronology represents the annual increments from 1975 to 2007 (33 years), with index values ranging from a low of 0.688 in 2006 to a high of 1.303 in 1977.

### Climate response analysis

**Figure 4:**
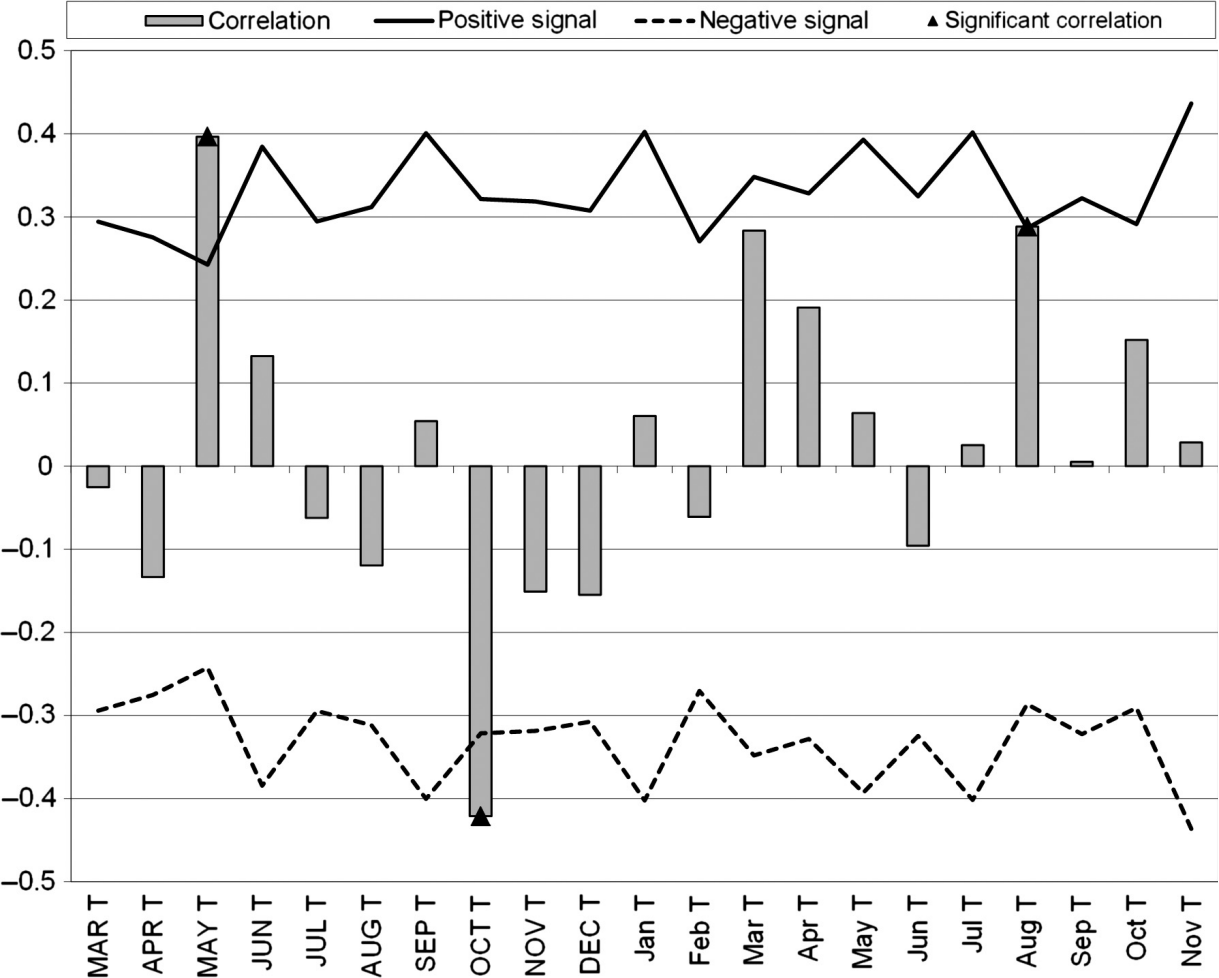
DENDROCLIM2002 correlation function analysis relating temperature to Blanding's turtle plastron growth increments. Months in capital letters represent previous year's growth, while months noncapitalized represent current year's growth. T, temperature. Positive and negative signal lines represent correlation thresholds.

**Figure 5:**
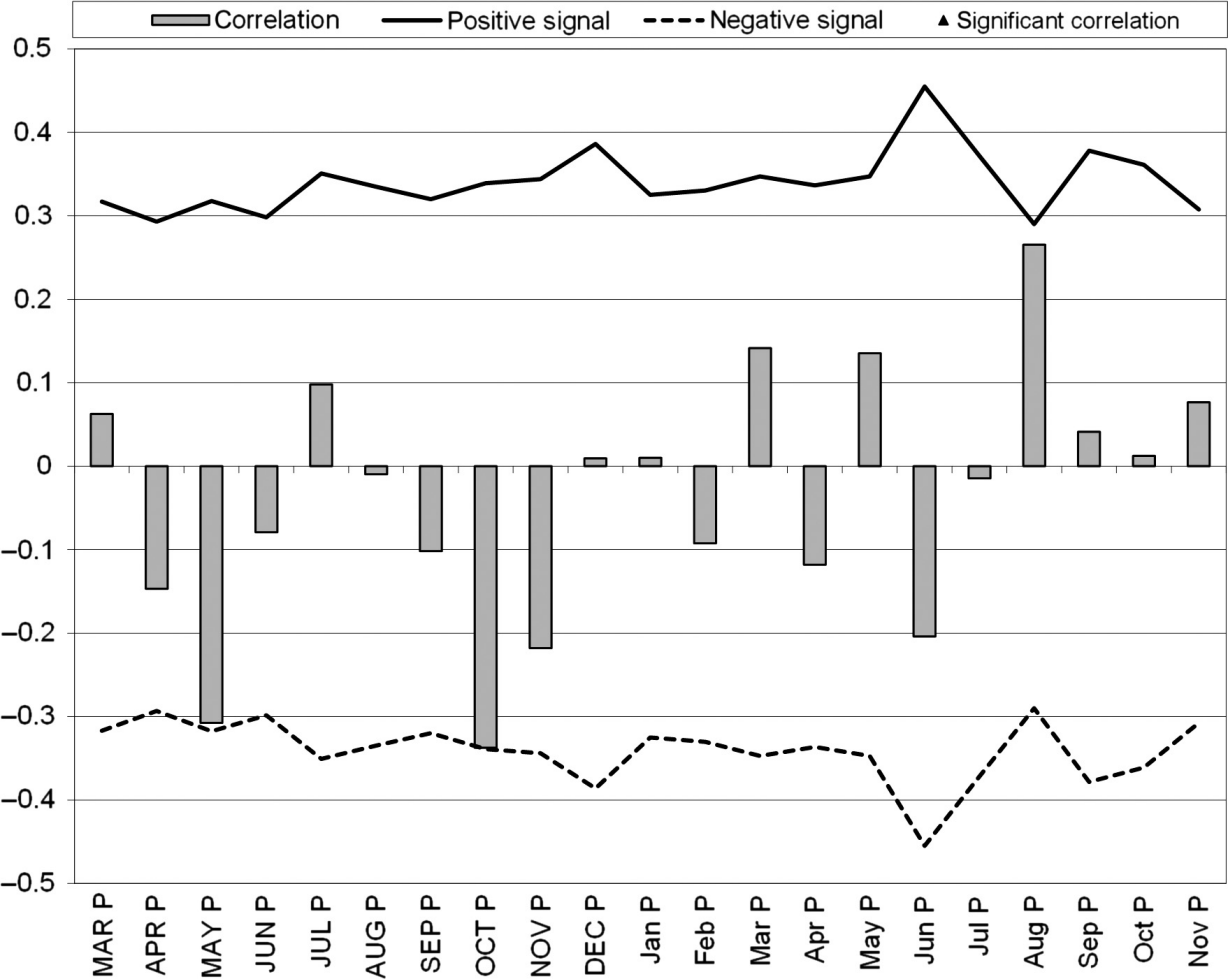
DENDROCLIM2002 correlation function analysis relating precipitation to Blanding's turtle plastron growth increments. Months in capital letters represent previous year's growth, while months noncapitalized represent current year's growth. P, precipitation. Positive and negative signal lines represent correlation thresholds.

Figures 3 and 4 illustrate the results of the DENDROCLIM2002 analysis for the relationship of temperature and precipitation to the turtle increment series. Plastron growth showed a positive correlation to the previous year's May temperature (*r* = 0.40; *P* = 0.02) and current year August temperature (*r* = 0.29; *P* = 0.05), as well as a negative correlation to the previous year's October temperature (*r* = −0.42; *P* = 0.01) (Fig. 4). While there was a general trend of a negative relationship, there was no significant relationship between Blanding's turtle growth increments and any month's precipitation (Fig. 5).

## Discussion

To make sure our data overlapped, it took many years to accumulate our measurement dataset (1998–2007), and our research has established that dendrochronology and dendroclimatology tools used for cross-dating, detrending, and climatic analysis can be used to study annual growth increments and environmental influences for the Blanding's turtle. Cross-dating was an indispensable step for our “cheloniochronology” analysis. It allowed us to identify and eliminate turtle patterns that did not pattern-match with the rest of those in the dataset, and correct increment dating errors (especially those from historical scans). Close examination of the electronic images for the individuals that required potential alignment changes or elimination revealed that many scans were dark, or of poor quality. Once brightness and contrast manipulations were applied to the images, dating errors were readily corrected. Some of the individuals eliminated from the dataset also had scute deformities that were not seen during the initial measurements.

COFECHA results indicated an “intermediate” mean sensitivity and series intercorrelation when used on a tree-ring scale (Grissino-Mayer 2001; in trees, values between 0.20 and 0.29 are considered intermediate for sensitivity), but with few other data published, it is difficult to understand whether this value has the same meaning for turtles. Our study showed a low autocorrelation when compared to tree studies (Grissino-Mayer 2001). The relatively low autocorrelation value found in our study would normally, for trees, indicate that there is little energy stored and carried over to initiate new growth in the following year. DENDROCLIM2002 results showed that there is a significant relationship between last year's temperature and this year's growth, leading us to conclude that the autocorrelation of 0.377 would not be considered low for this population of Blanding's turtle. A study on the plastron increments of preserved and live wood turtles (*Glyptemys insculpta*) by Robichaud (2006) revealed that this turtle's growth increments were pattern-matched with a slightly higher intercorrelation (0.540), mean sensitivity (0.319), and autocorrelation (0.589). As a relative comparison, these values may illustrate that the Blanding's turtle has intermediate autocorrelation, while the wood turtle has high autocorrelation. However, both this and Robichaud's study have limited sample size and depth. A greater sample depth of older immature turtles in this study (e.g., 25-year-olds) would likely increase the overall intercorrelation and autocorrelation. The stored reserves required for the Nova Scotia Blanding's turtle may be needed during overwintering processes, but the carryover was considerably lower than for most tree species in the region, which enter full dormancy (Robichaud and Laroque 2008, Trindade et al., 2011).

Our master chronology illustrates no significant trend throughout the time series. This is corroborated by the intermediate mean sensitivity value and lower autocorrelation value; both suggest that most of the incremental growth of Blanding's turtle seems to be influenced by environmental parameters in a given year. This point seems to be more valid where the chronology has a higher sample depth, compared with some of the oldest values (e.g., 1976), where the sample depth included only one turtle. The climate analysis indicated that the current year's August temperature positively influenced plastron growth; this is likely related to food abundance and storage of reserves before overwintering. This result also indicates that growth in immature turtles' current growth year is mostly influenced late into the active season. Positive correlation to the previous year's May temperature likely links to the early flush of primary productivity in the turtle's habitat leading to a better foraging year overall and indicates that the past year's early emergence by the turtle positively influences the present year's growth. This result was consistent with findings in wood turtle populations in neighboring New Brunswick (Robichaud 2006). The negative relationship to previous year's October temperature likely relates to the turtle's overwintering behavior. Hotter weather would delay overwintering and use reserves stored in August at an earlier than ideal time. While the analysis on precipitation was not statistically significant, the short-term comparison of the time span of our dataset could be influencing this result. Previous October precipitation was very nearly significantly negative in influencing Blanding's turtle growth (Figs 4 and 5) and could be a result of nonideal conditions during movements to overwintering areas (Newton and Herman 2009).

With a larger dataset and the application of commonly used temperature models, conservation biologists should be able to predict how climate change will impact the growth and size of the Blanding's turtle in Nova Scotia, which in turn impacts physiological functions such as clutch size (McNeil 2002) and immature turtle survival (McMaster and Herman 2000; Arsenault 2011). Understanding the influence of climate variables is particularly important for species with already low numbers and those at their northern range limits, where even small changes in climate may have significant impacts.

Blanding's turtle plastron growth increments can be cross-dated to build one common chronology revealing average, good, and poor years of growth. Our hypothesis that this turtle's growth is related to climate variables was statistically supported for the variable of temperature but not for the variable of precipitation. It is of interest to ecological and conservation science to attribute specific months' temperature as positive or negative drivers of Blanding's turtle growth. We confirmed that growth is influenced by the last year's conditions, as predicted using previous year's May and October average monthly temperatures, and it is also influenced by the current year's August average temperature.

Utilizing dendrochronology methods and software should be adaptable by other researchers wanting to explore the influence of environmental variables on turtles. Understanding these variables will help researchers better understand ecological needs, and how to protect resources also influenced by these variables for all turtles at their northern range limits, where climate changes may show more pronounced impacts.
